# Supplementary material for: 3D Bone Morphology Alters Gene Expression, Motility, and Drug Responses in Bone Metastatic Tumor Cells
Source: Int J Mol Sci. 2020 Sep 21;21(18):6913. doi: 10.3390/ijms21186913 (PMC7555977; doi:10.3390/ijms21186913)
Supplement: Supplementary file 1 [file ijms-21-06913-s001.zip › ijms-929881-supplementary/3D Tumor_Supplementary Data_Revised 9.16.docx]

**Supplementary Methods**

*Cell culture.*

Patient-derived chondrosarcoma cells were used for testing viability of primary cells on the 3D scaffolds. Primary chondrosarcoma cells were isolated from specimens obtained from the Cooperative Human Tissue Network (CHTN) Western Division in accordance with the Institutional Review Board (IRB)-approved protocol and upon informed consent from patients undergoing surgical resection. Dedifferentiated chondrosarcoma tissue was removed from the left distal femur of 67 y/o male who had not received chemo or radiation. For cell isolation from surgical specimen, fresh tissue was minced into small pieces and incubated with 0.25% trypsin in HBSS overnight at 4°C. Dissociated pieces were then strained with a 70 μm filter and washed with PBS. Recovered cells were cultured in RPMI 1640 media (Corning Cellgro, Manassas, VA).

For molecular inhibition experiments, MDA-MB-231 cells were stably transfected with the following: (i) a targeted shRNA for ITGB3 (shβ3) (Santa Cruz) or a control plasmid (Santa Cruz) using an shRNA transfection reagent (Santa Cruz) per manufacturer's instructions; (ii) a Gli2-Repressor construct (Gli2 EnR) or (iii) a mutant TGF-β receptor type II construct (rII ΔCyst) using Lipofectamine transfection reagent and Plus reagent (Invitrogen), per manufacturer’s instruction. Cells were plated onto rigid 2D films (3.6x10^5^ tumor cells per film) or 420R 3D scaffolds (0.5x10^6^ cells/scaffold). After 48 hours of culture, RNA was harvested for qRT-PCR analysis.

Chondrosarcoma cells were cultured in RPMI 1640 media and transfected MDA-MB-231 cells were cultured in DMEM (Corning Cellgro, Manassas, VA). All media was supplemented with 10% fetal bovine serum (Peak Serum, Colorado, USA) and 1% penicillin-streptomycin (Corning Cellgro, Manassas, VA).

*Cell viability assay.*

For viability time course studies, 0.5x10^6^ patient derived chondrosarcoma cells and 1x10^6^ MDA-MB-231 cells were seeded on 3D scaffolds and cultured for 7 and 2 days respectively. Scaffold-adhering cells were detached using accutase (EMD Millipore) and trypan blue was used to determine cell viability on 3D scaffolds on 1, 3, 5, and 7 days for patient derived chondrosarcoma and 2 days for the MDA-MB-231 cells. Each experiment was repeated three times. n=3 replicate measurements.

*Immunofluorescence*

For analysis of adhesion proteins, Focal Adhesion Kinase (FAK) or Integrin Beta 3 (Iβ3), 560C, 420C, 560R, and 420R scaffolds were seeded with 0.5x10^6^ MDA-GFP cells and cultured for 24 hours. After 24 hours, scaffolds were harvested and fixed in 10% formaldehyde for 30 minutes at room temperature. Scaffolds were washed with PBS, incubated in a 3% BSA blocking solution for 1 hour, and incubated overnight at 4°C in anti-FAK primary antibody (1:800, Cell Signaling #3285) or Alexa 488-anti-Iβ3 antibody (1:500, SantaCruz #sc-365679). An Alexa Fluor® 546-tagged secondary antibody was used for detection of FAK. Representative 40x images were taken on an Olympus BX41 upright microscope. Number of focal adhesions per cell were measured using Metamorph Image analysis software.

*Tumor Metastatic Array*

TaqMan™ Array Human Tumor Metastasis 96 well plate (ThermoFisher, Waltham, MA, USA) was used to determine tumor metastatic gene expression as per manufacturer’s protocol. Briefly, total RNA was purified from *in vivo* MDA-MB-231-GFP cells seeded 3D scaffold samples and reverse transcribed to cDNA using the TaqMan® Reverse Transcriptase Kit (Applied Biosystems™, Waltham, MA, USA). cDNA was added to TaqMan™ Array Human Tumor Metastasis 96 well plate and gene expression was quantified relative to four endogenous genes present in array panel.

*Immunohistochemistry*

Immunohistochemical (IHC) staining was performed on sections of excised 3D scaffolds to evaluate the expression of CD11b (1:1000, Abcam EP1345Y), F4/80 (1:1000, BioRad MCA497EL), Ly6G (1:1000, Abcam ab25377), Ki67 (0.1 μg/mL, Abcam ab15580 ), and Caspase 3 (1:1000, Cell Science technology #9662 ). Briefly, antigen retrieval was performed with citrate buffer followed by blocking in 5% goat serum for 1h and primary antibody incubation overnight at 4^0^C. Sections were incubated in an anti-rabbit secondary antibody (Santa cruz, #sc2004) for 2h at room temperature and detected with NovaRed peroxidase kit (Vector Labs). % stained area over total area was quantified using Metamorph imaging analysis software or ImageJ software.

**Supplementary Figures**

**
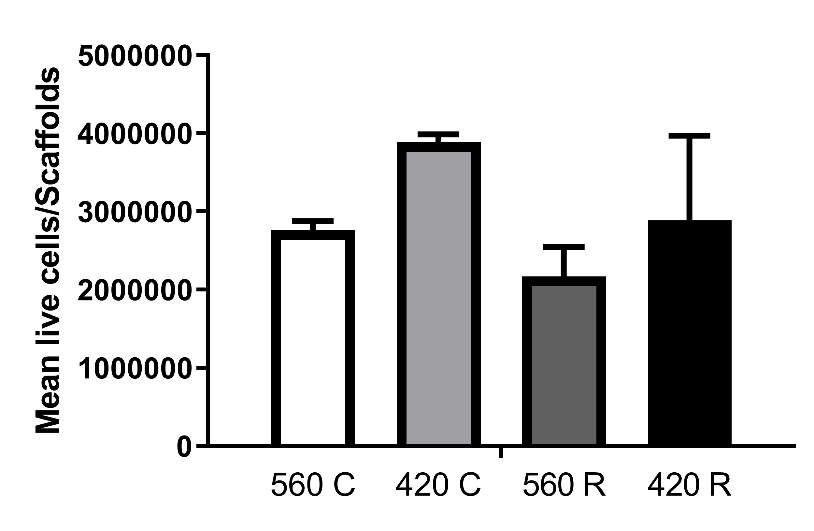
**

**Figure S.1. Rigidity and pore size do not affect viability of MDA-MB-231 cells on 3D scaffolds.** Viability of MDA-MB-231 cells on 3D scaffolds, measured by Trypan blue, showed no statistical differences between 560C, 420C, 560R, and 420R 3D t-FDM scaffolds after 48 hours in culture.

**
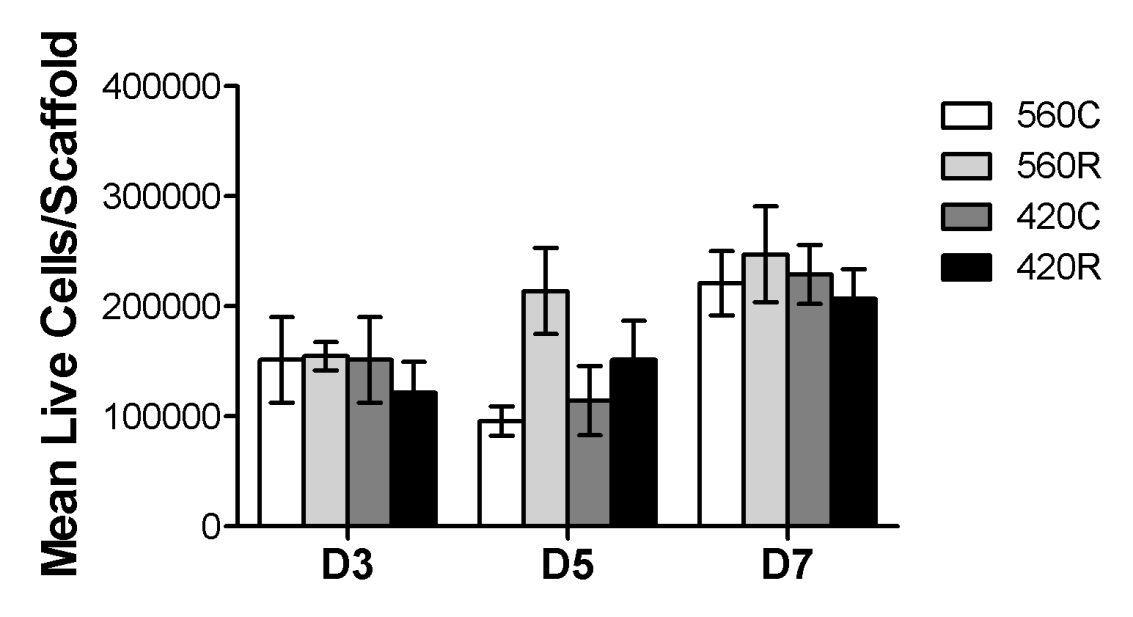
**

**Figure S.2. Rigidity and pore size do not affect viability of patient derived chondrosarcoma cells on 3D scaffolds.** Viability of patient derived chondrosarcoma cells on 3D scaffolds, measured by Trypan blue, showed no statistical differences between 560C, 420C, 560R, and 420R 3D t-FDM scaffolds over a time period (days – D3, D5 and D7).


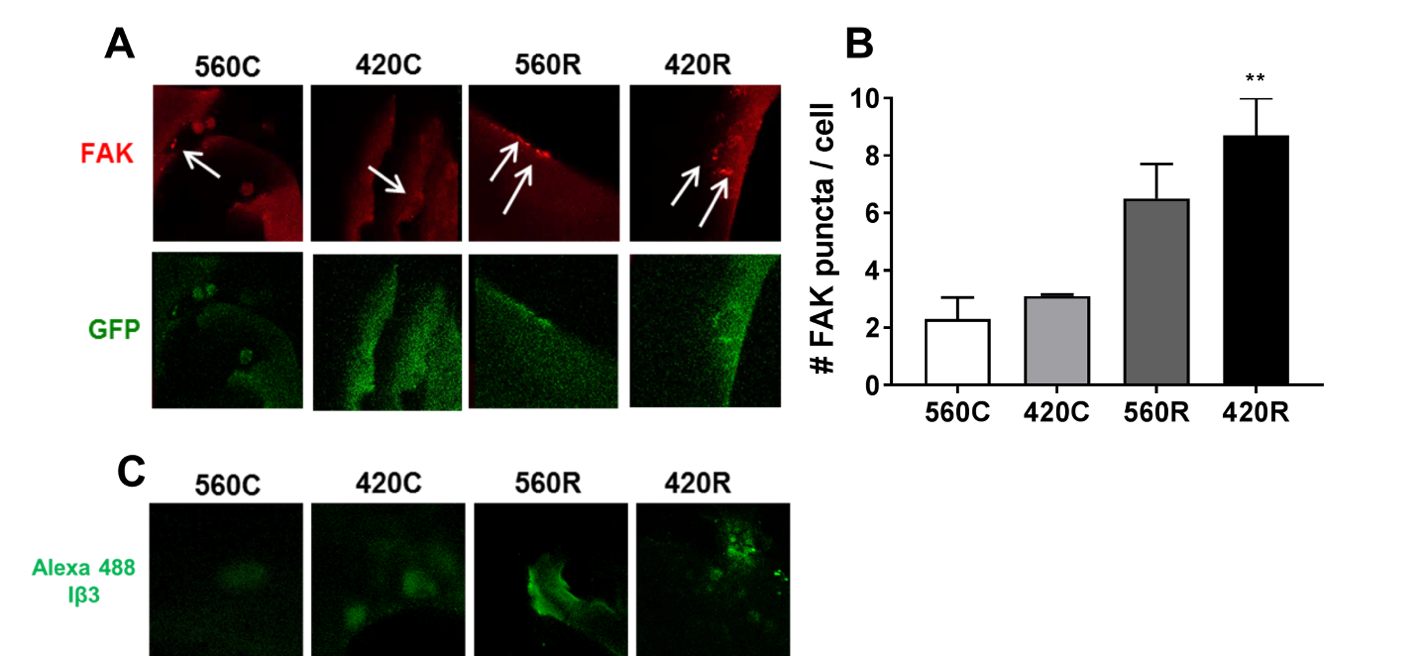


**Figure S.3. Effect of rigidity and pore size on cell adhesion.** (**A**) Representative microscopy images of Focal Adhesion Kinases (FAK, Red, top row) in cells (GFP, green, bottom row) grown on 560C, 420C, 560R, and 420R scaffolds. (**B**) Quantification of number of Focal Adhesions / cell for cells grown on 560C, 420C, 560R, and 420R scaffolds. (**C**) Representative microscopy images of Integrin Beta 3 (Iβ3) in cells grown on 560C, 420C, 560R, and 420R scaffolds.

**
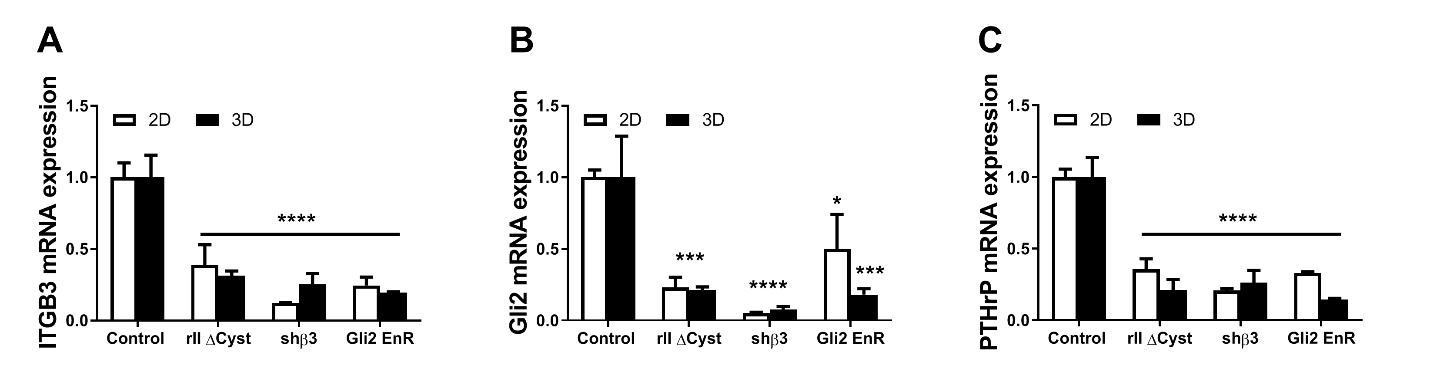
Figure S.4. Effect of TGFβ, Integrin β3, and Gli2 Inhibition on bone-metastatic gene expression in 3D culture.** MDA-MB-231 cells transfected with a mutant TGFβ receptor type II (rII ΔCyst), shRNA for Integrin β3 (shβ3), or a repressor domain of Gli2 (Gli2 EnR) were seeded onto rigid 2D films and 420R 3D scaffolds and cultured under static conditions for 48 hours. mRNA expression was measured by qRT-PCR. (**A**) *ITGB3*, (**B**) *Gli2*, and (**C**) *PTHrP* expression significantly decreased in both 2D and 3D culture conditions. Data presented as fold change over untreated control. * p<0.05, ***p<0.001, ****p<0.0001

**Figure S.5** Representative *in vivo* immunohistochemistry images of scaffolds and quantification of Ki67 and Caspase3 staining. Images show scaffold pores have tissue integration as confirmed by Hematoxylin staining, and positive immunohistochemical staining (dark brown staining). (**A**) 20x images and (**B**) quantification of Ki67 in 3D scaffolds show increased cell proliferation in 420R compared to 560C. (C) 20x images and (D) quantification of Caspase3 reveal relatively low apoptosis occurring in tumors grown in 3D scaffolds *in vivo*. Significantly higher % Casp3 staining was found in 560R compared to 560C. Compliant vs Rigid: *p<0.05. 560 vs 420: +p<0.05.


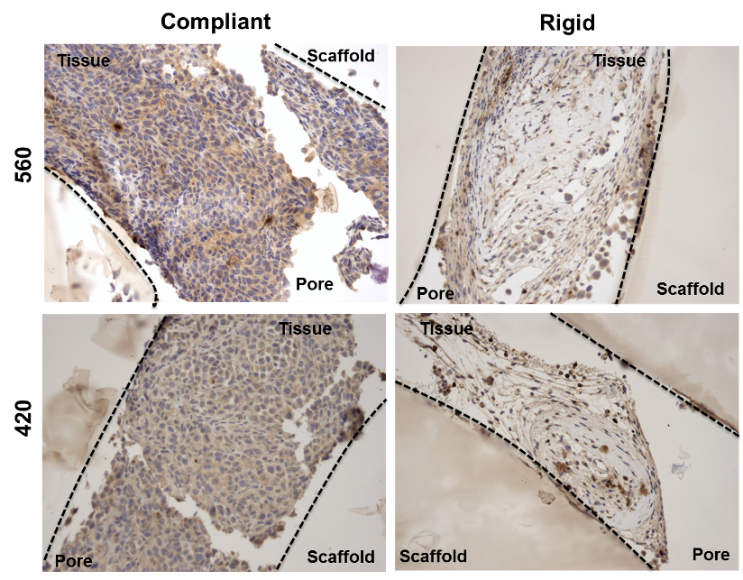


**Figure S.6** Representative *in vivo* immunohistochemistry images of scaffolds stained for ly6G by immunohistochemistry. Images show scaffold pores have tissue integration as confirmed by Hematoxylin staining, and positive immunohistochemical staining (dark brown staining).

**Supplementary Movie Legends**

**Movie S.1 Live cell imaging on 560C 3D scaffolds.** Compliant 3D scaffolds with a 560 μm pore size (560C) were coated with fibronectin and seeded with 5x10^5^ MDA-MB-231-GFP cells. Cell seeded scaffolds were placed in a live cell chamber, cultured at 5% CO_2_ and 37°C, and monitored by light microscopy for 48 hours. Images of the same field were taken every hour for 48 hours. Single cell movement was analyzed by ImageJ software. N=3 biological replicates.

**Movie S.2 Live cell imaging on 420C 3D scaffolds.** Compliant 3D scaffolds with a 420 μm pore size (420C) were coated with fibronectin and seeded with 5x10^5^ MDA-MB-231-GFP cells. Cell seeded scaffolds were placed in a live cell chamber, cultured at 5% CO_2_ and 37°C, and monitored by light microscopy for 48 hours. Images of the same field were taken every hour for 48 hours. Single cell movement was analyzed by ImageJ software. N=3 biological replicates.

**Movie S.3 Live cell imaging on 560R 3D scaffolds.** Rigid 3D scaffolds with a 560 μm pore size (560R) were coated with fibronectin and seeded with 5x10^5^ MDA-MB-231-GFP cells. Cell seeded scaffolds were placed in a live cell chamber, cultured at 5% CO_2_ and 37°C, and monitored by light microscopy for 48 hours. Images of the same field were taken every hour for 48 hours. Single cell movement was analyzed by ImageJ software. N=3 biological replicates.

**Movie S.4 Live cell imaging on 420R 3D scaffolds.** Rigid 3D scaffolds with a 420 μm pore size (420R) were coated with fibronectin and seeded with 5x10^5^ MDA-MB-231-GFP cells. Cell seeded scaffolds were placed in a live cell chamber, cultured at 5% CO_2_ and 37°C, and monitored by light microscopy for 48 hours. Images of the same field were taken every hour for 48 hours. Single cell movement was analyzed by ImageJ software. N=3 biological replicates.

**Supplementary Tables**

| **Gene Expression** | **Gene ID** | **Fold Change** |
| --- | --- | --- |
| connective tissue growth factor | CTGF | 1.12 |
| chemokine (C-X-C motif) receptor 4 | CXCR4 | 1.9 |
| interleukin 11 | IL11 | 1.321 |
| matrix metallopeptidase 9 | MMP9 | 3.34 |
| Secreted phosphoprotein | OPN (SPP1) | 0.892 |

**Table legend**

**Table S.1 Metastatic panel in 3D t-FDM scaffolds *in vivo***. MDA-MB-231-GFP seeded scaffolds 3D scaffolds (420R and 420C) were harvested. Gene expression was determined using tumor metastatic array. Fold change was measured as the ratio of gene expression of tumor cells seeded on rigid and compliant 3D scaffolds (420R/420C).
